# Optimization of the ASPIRE Spherical Parallel Rehabilitation Robot Based on Its Clinical Evaluation

**DOI:** 10.3390/ijerph18063281

**Published:** 2021-03-22

**Authors:** Paul Tucan, Calin Vaida, Ionut Ulinici, Alexandru Banica, Alin Burz, Nicoleta Pop, Iosif Birlescu, Bogdan Gherman, Nicolae Plitea, Tiberiu Antal, Giuseppe Carbone, Doina Pisla

**Affiliations:** 1 CESTER, Technical University of Cluj-Napoca, 400641 Cluj-Napoca, Romania; paul.tucan@mep.utcluj.ro (P.T.); ionut.ulinici@omt.utcluj.ro (I.U.); alexandru.banica@omt.utcluj.ro (A.B.); alin.burz@mep.utcluj.ro (A.B.); nicoleta.pop@mep.utcluj.ro (N.P.); iosif.birlescu@mep.utcluj.ro (I.B.); bogdan.gherman@mep.utcluj.ro (B.G.); nicolae.plitea@mep.utcluj.ro (N.P.); tiberiu.alexandru.antal@mep.utcluj.ro (T.A.); 2 DIMEG, University of Calabria, 87036 Cosenza, Italy; giuseppe.carbone@unical.it

**Keywords:** robotic assisted rehabilitation, neuro-muscular disorders, stroke clinical trial, design, optimization

## Abstract

The paper presents the design optimization of the ASPIRE spherical parallel robot for shoulder rehabilitation following clinical evaluation and clinicians’ feedback. After the development of the robotic structure and the implementation of the control system, ASPIRE was prepared for clinical evaluation. A set of clinical trials was performed on 24 patients with different neurological disorders to obtain the patient and clinician acceptance of the rehabilitation system. During the clinical trials, the behavior of the robotic system was closely monitored and analyzed in order to improve its reliability and overall efficiency. Along with its reliability and efficiency, special attention was given to the safety characteristics during the rehabilitation task.

## 1. Introduction

One of the most common neurological diseases of our century is stroke [1]. Globally, death caused by stroke has an incidence of 11.8%, coming in second after ischemic heart disease with an incidence of 14.8% [2]. Statistics provided by the European Society of Cardiology in 2019 show an average stroke prevalence in Europe of 1276 strokes/100,000 inhabitants, with the lowest prevalence in Italy (570 strokes/100,000 inhabitants) and highest in Latvia (1869 strokes/100,000 inhabitants), regardless of gender [3].

There are a series of consequences that can occur after a stroke. Most common are post-stroke depression (PSD), vascular cognitive impairment (VCI), and post-stroke fatigue (PSF) [4]. Stroke is deadly for about 20% of cases, leading to 80% of cases where survivors might need post-stroke special care that implies the involvement of specialized personnel, since most of survivors suffer a loss of mobility, impaired speech, or cognitive problems [5]. With more than 80 million stroke survivors worldwide, providing special care able to aid all these survivors is foreseen to be one of the main challenges of the 21st century. The forecast provides data according to which, in 2030, 4% of adults will suffer a stroke, and the annual medical cost of stroke will increase from 71.55 billion USD in 2012 to 183.13 billion USD in 2030, only in the USA [6]. Most of stroke survivors suffer a different type of limb impairment that requires a certain type of physical rehabilitation that can sometimes take up to two years, depending on the physical condition of the stroke survivor and the impairment severity.

All the above data predicts a future crisis around 2030 when the medical system will be unable to provide the specific care for each stroke survivor because of the continuous increase in the number of stroke patients correlated with the aging of population. It is foreseen that the population aged over 65 will achieve 30% of the world population in 2060 [7]. To prevent this collapse, a change in the paradigm is needed, meaning that patient management must be performed in other ways that would allow physical therapists to work with more patients at the same time while providing personalized efficient treatment programs that will improve their outcomes in a lower time span.

Since 1990, robotic-assisted medical rehabilitation has become a valuable solution in overcoming the qualified personnel shortage from the medical system [8]. The physical rehabilitation of stroke survivors left with limb impairments is performed by a clinician using repetitive motions of the disabled limb in order to rebuild the neuronal paths lost during the stroke. These repetitive motions can be easily performed with the help of a robotic system under the supervision of a kinetotherapist. Furthermore, a robotic system can integrate additional stimuli that contribute to a faster recovery: visual and audio interactive tools, personalization, a real-time sensor system, etc.

The advancements in kinematics and control [9,10,11,12,13,14,15,16,17,18,19] have led towards safer robot behaviors, enabling the development of multiple robotic solutions for the rehabilitation of the upper limbs [20,21,22,23,24,25,26], some of them being developed until the stage of clinical trials.

In 2009, Kai et al. [27] performed a clinical study using the MIT-Manus robotic rehabilitation system. During the experiment, the brain signals were acquired using a BCI (Brain–Computer Interface) system, and the affected limb of the patient was strapped to the robotic system. Eighteen patients were selected for the clinical evaluation of the robotic system that were able to commit for 12 sessions of rehabilitation. The results of the study revealed greater motor improvements in the robotic-assisted rehabilitation than the classic rehabilitation, but the results were considered inconclusive due to large variations in the motor improvements and the limited number of patients recruited for the study.

In 2015, Fong et al. [28] performed a clinical evaluation of the ArmeoPower robotic system using 10 healthy subjects aged between 22 and 34 years old. The rehabilitation procedure was divided into five sessions (Free Reaching, 3x Robot Reaching, Free Reaching), where each subject completed a reaching task 120 times using their dominant hand. The study revealed an evolution of the classical movement metrics; however, for closer to reality results, a larger number of subjects should be used with different neurological diseases.

In 2021, DeBoon et al. [29] proposed a nine DOFs (Degrees of Freedom) robotic system for the rehabilitation of the upper limb. The redundant robotic system consists of nine revolute active joints, and it is the first nine DOF robot for upper limb rehabilitation. The advantage of the robotic system is the capability to provide complex rehabilitation trajectories, but controlling a robotic system with many moving parts requires a solid dynamic control throughout the entire rehabilitation procedure to ensure the safety characteristics of the procedure. In Table 1, a series of robotic devices for upper limb rehabilitation are described in terms of the targeted area, DOFs, therapy type, architecture characteristics, and development stage.

A wide number of upper limb rehabilitation robotic solutions consist of exoskeletons [40,41]. These solutions have the advantage of allowing complex motions due to joint constraints and facilitate a faster and natural neuro-recovery due to the functional range of motion. The disadvantages of these robotic solutions are the price of the system—each exoskeleton must be uniquely designed to adapt patient anthropomorphic characteristics—and the fact that the entire weight of the robotic system is carried by the patient; at the same time, the solution is accessible only to patients that have regained some of the motoric capabilities of the impaired limb.

Among the robotic solutions for the rehabilitation of the upper limb, some platforms were designed and tested regarding the environment of the rehabilitation task, and using virtual reality, the rehabilitation procedure was placed in an environment more stimulating for the patient.

Saposkik et al. [42] conducted in 2010 a pilot study regarding virtual reality (VR) in stroke rehabilitation. The study included two parallel groups of stroke patients, and it had a time span of two months. During the study the feasibility, safety, and efficacy of the rehabilitation using virtual reality was compared against the recreational therapy (playing cards, bingo, etc.). The average age of the patients involved in the study was 61.3, and the average time spent in the recreational rehabilitation was 388 min, while the time spent in the virtual reality rehabilitation was 364 min. The study revealed no significant differences between the virtual-environment rehabilitation and the conventional rehabilitation. The study highlighted the potential of virtual reality gaming used as means to provide stroke rehabilitation, but it remains an unproved treatment, and more studies are required to prove the efficacy of the treatment.

Some other studies recorded proof of the efficiency of a virtual environment used in stroke rehabilitation. Estapa et al. [43] used a Kinect-based exergaming system for the rehabilitation of patients with neurological disorders (2016), Munoz et al. [44] proposed an interactive gaming-driven rehabilitation of the upper limbs (2019), and Bai et al. [45] also proposed a home-based multi-scene system for the rehabilitation of post-stroke patients that was able to simulate fishing activities, parkour, activities of daily living, and virtual walks.

Given the above solutions for post-stroke rehabilitation, a valuable solution should include both aspects: a robotic system and virtual environment to provide significant improvements in the quality of life of stroke survivors. Based on the already proven paradigm that patients that are focused on the task during rehabilitation recover faster, the development of additional stimuli like VR and interactive games can have benefits, as long as they succeed in addressing the needs and interests of the patient.

The focus of this paper is on the design optimization of a spherical parallel robot for shoulder rehabilitation. The optimization is a two-stage process. The first stage is the preclinical evaluation, where the design of the robotic structure is improved based on the clinicians’ feedback before performing clinical trials. The second stage improvements are based on the clinicians and patients’ feedback based on the clinical trials. In the second section of the paper, the ASPIRE robotic structure is presented in terms of the initial experimental model and control system, and the planning of the clinical trials and the experimental tests using patients are described. The third section of the paper presents the results obtained and design optimization of the robotic system, followed by the discussions and conclusions.

## 2. Materials and Methods

### 2.1. The Experimental Model of ASPIRE

ASPIRE is a spherical parallel robotic system that targets the adduction, abduction, flexion, and extension rehabilitation motions of the shoulder joint and the pronation and supination of the forearm [46]. The following section is subdivided into two parts in order to separate the features of mechanical structure from the ones of the control system.

#### 2.1.1. Mechanical Structure of ASPIRE

**Figure 1:**
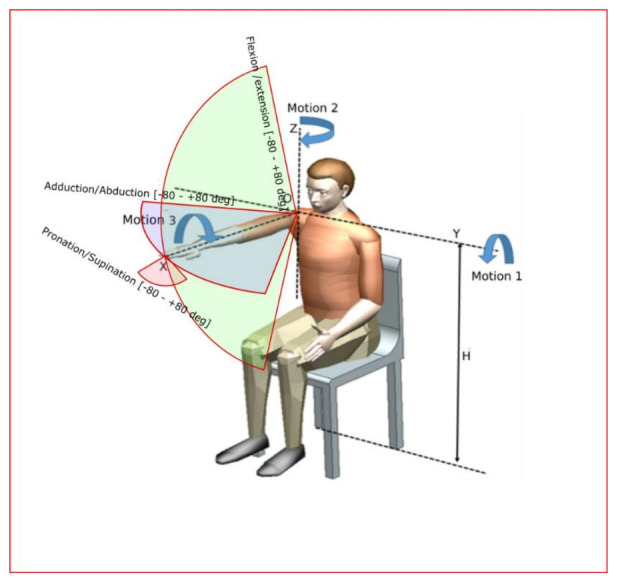
The targeted motion of ASPIRE [24].

The main design feature of the robotic system consists of two circular guides that lead to a spherical motion of the characteristic point of the mechanism. Starting from the concept stage, the patient is embedded in the design of the robotic structure, and the center of the sphere given by the two circular guides is placed in the center of the patient shoulder, allowing the entire arm motion with respect to the shoulder rehabilitation motion. The targeted motions of ASPIRE are flexion/extension of the shoulder with a motion range of +/− 80 degrees, adduction/abduction of the shoulder with a motion range of +/− 80 degrees, and pronation/supination of the forearm with a range of +/− 80 degrees (Figure 1 [24]).

**Figure 2:**
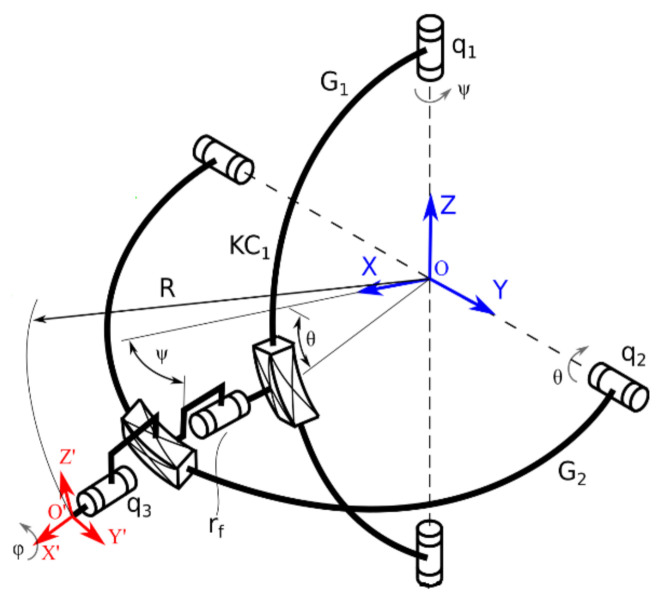
The kinematic scheme of ASPIRE.

The kinematic scheme of the ASPIRE robot is presented in Figure 2. The reference system OXYZ is placed at the center of the shoulder articulation. G1 is the vertical circular guide and G2 is the horizontal circular guide. The characteristic point placed in the reference system O’X’Y’Z’ moves on a sphere of radius R. The active joint of the mechanism are q_1_, q_2_ and q_3._ Ψ, θ and φ represents the angular displacement of the reference characteristic point with respect to the reference system OXYZ. The equations of the active joints are given in Equations (1)–(3).

(1) $$q_{1}=atan2\left(\sin\;\psi ,\cos\psi \right)$$

(2) $$q_{2}=atan2\left(\sin\;\theta ,\cos\theta \right)$$

(3) $$q_{3}=\varphi$$

**Figure 3:**
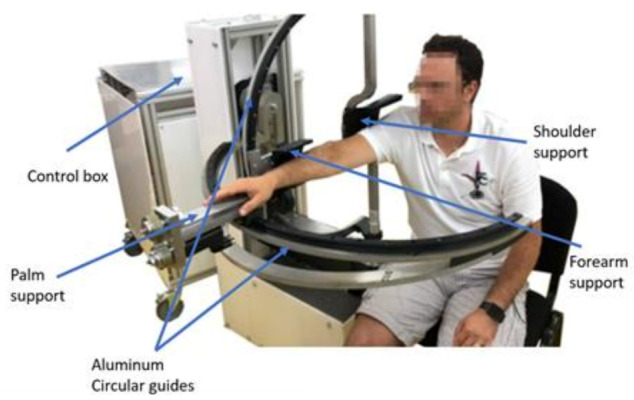
Initial experimental model of ASPIRE.

The initial experimental model of ASPIRE is presented in Figure 3. Along with the kinematic elements needed for the rehabilitation motion of the shoulder, other aiding elements were added. The frame of the robotic structure was made of aluminum profiles, and the case of the robot was made of Plexiglas. The circular guides were manufactured from aluminum, and the sliding carriages were 3D-printed and properly greased to ensure a low friction coefficient. The shoulder rest was also 3D-printed in such a manner to ensure the support of the shoulder during the flexion and extension motions. The forearm support was 3D-printed and embedded into an adjustment mechanism to fit different lengths of the forearm. The palm rest was made of aluminum and embedded into the pronation/supination mechanism. The connection between the vertical circular guide and the horizontal one was made using a passive revolute joint between the circular guides. An actuated height adjustment mechanism was added to allow easy setup of the robot between patients of different heights.

For easy acceptance from the patient, the actuating mechanisms were embedded in the Plexiglas case, and only the moving elements that come into direct contact with the patient for performing the rehabilitation motion were visible. For easy maneuverability, the entire robotic structure was equipped with locking wheels.

#### 2.1.2. The Control System of ASPIRE

**Figure 4:**
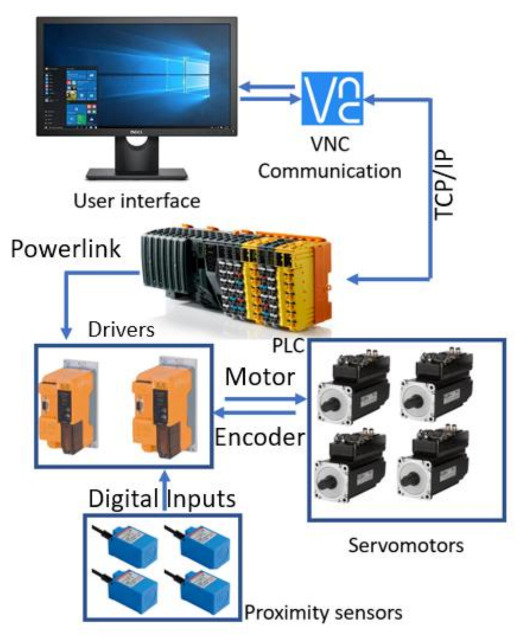
Control architecture of ASPIRE. PLC: Programable Logical Computer and VNC: Virtual Network Computing.

The control architecture of ASPIRE is presented in Figure 4.

**Figure 5:**
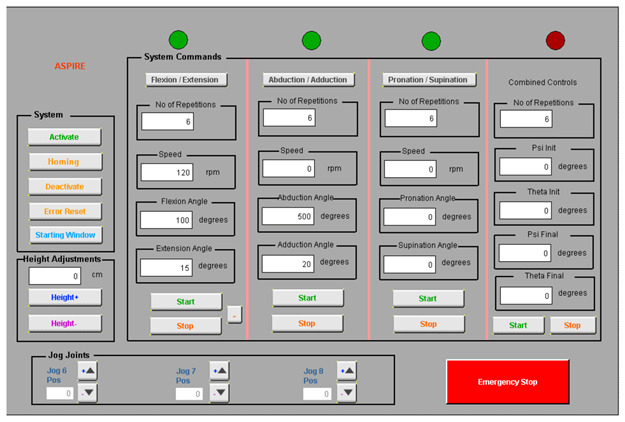
Graphical User Interface of ASPIRE.

The entire rehabilitation procedure is controlled through a graphical user interface (Figure 5) that communicates with the Programable Logical Computer (PLC) of the robotic system through Virtual Network Computing (VNC). The PLC controls the two drivers of the system that ensure the functioning of four servomotors and receives data from four proximity inductive sensors.

**Figure 6:**
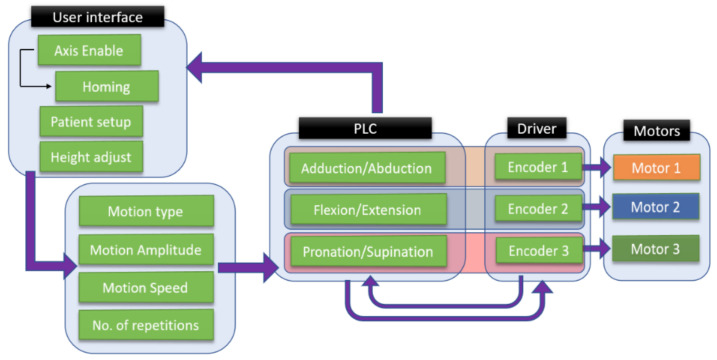
Control strategy of ASPIRE.

The control strategy of the robotic system is presented in Figure 6. The setup of the robotic system is made using the user interface, where the first step is to power up the motors and the initialization of the motion axes. After the robotic system is initialized, the patient setup is performed, and the height of the rehabilitation device is adjusted to the height of the patient. The user interface allows the selection of the rehabilitation motion, and the motion parameters are given by the kinetotherapist and input into the interface in terms of the amplitude, speed, and number of repetitions.

The rehabilitation motion parameters are parsed into the PLC of the robotic system, which identifies the rehabilitation motion and sends the motion parameters to the drivers that control each motor of the robotic structure. The hardware architecture (Figure 4) presents a number of 4 servomotors, while the control strategy figure (Figure 6) shows only 3 motors due to the fact that the fourth motor does not intervene in the rehabilitation procedure; it is used to adapt the height of the robotic structure to the height of the patient.

### 2.2. Clinical Trials

Performing the clinical trials was one of the final steps of the robotic system validation. Before performing tests with patients, the robotic system underwent a lab testing phase to check the safety of the user during the rehabilitation procedure. Additionally, before any tests were performed, a medical rehabilitation protocol was developed and successfully used during the lab tests and clinical trials.

#### 2.2.1. Lab Validation and Rehabilitation Protocol

**Figure 7:**
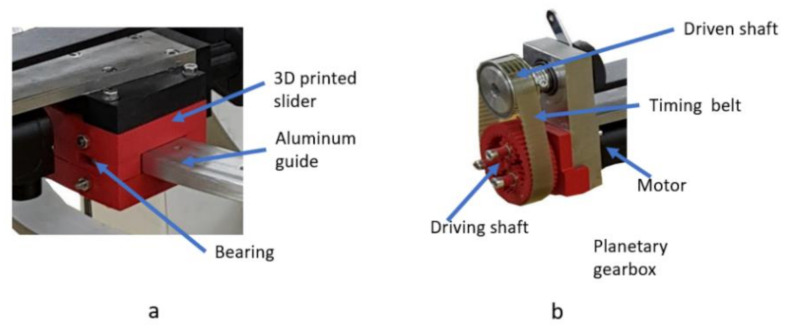
Optimization after functionality tests: (**a**) friction reduction and (**b**) planetary gear.

Before the clinical trials, a series of in-lab tests were performed with healthy subjects to validate the functionality of the robotic system. During the functionality tests, the 3D-printed circular guides proved to be inefficient, introducing some unwanted vibrations into the mechanical system, so the guides were redesigned to embed rolling bearings to reduce the friction coefficient (Figure 7a). During the functionality test, the motor for the pronation/supination of the forearm proved to be unable to carry the mass of the limb, so a special planetary gear [47,48,49,50] with a reductio ratio of 1:11 (Figure 7b) was designed and manufactured using a 3D printer.

**Figure 8:**
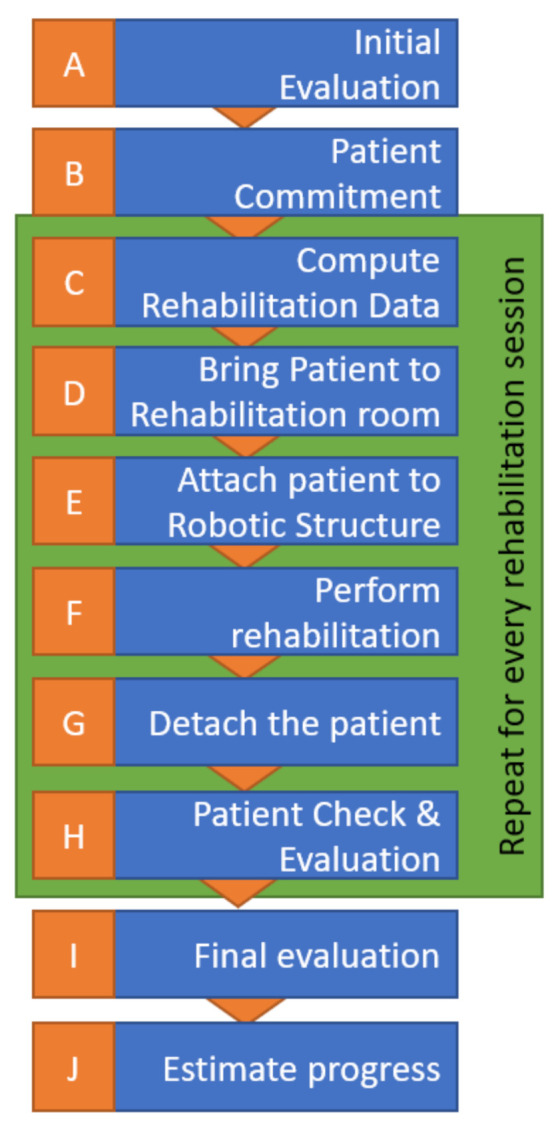
Medical rehabilitation protocol flow chart.

A specific robotic rehabilitation protocol was required to perform the clinical trials [51], which was defined and then experimentally validated by medical experts. The protocol is graphically represented in Figure 8 and detailed below.

- When admitted to the hospital, the patient should go through an initial assessment performed by a physical therapist and receive an initial score indicating his impairment levels for the upper limb before rehabilitation treatment. The data is recorded by the therapist for future analysis and for the setup of the starting motion parameters for the robot (evolution/involution record). A rehab program is defined for 7–14 days with one or two daily sessions.
- Before starting the rehabilitation, the involved patient must sign an informed consent and be conscious and stable hemodynamically, present no fever symptoms, and the brain lesion confirmed via CT (Computer Tomograph) or MRI (Magnetic Resonance Imaging) to exclude other diseases than the neurologic one.
- The data recorded in a previous stage are used to compute the robot motion amplitudes for the first rehabilitation session. For the next sessions, a daily increase of the amplitudes is applied in the range of 5–10%, aiming to reach the targeted amplitude around day 5.
- The patient will be caried to the robotic rehabilitation room by a stretcher-bearer using a wheelchair or walking, depending on the health state of the patient.
- The patient is strapped into the robotic device (the height of the robotic device is adjusted, and the forearm adjustment mechanism is adapted to the patient).
- The robotic rehabilitation procedure is performed based on the therapist’s recommendations using a predefined exercise of 3 × 10 repetitions for each rehabilitation motion.
- After the predefined exercises are performed, the robotic device is detached from the patient.
- The patient undergoes a physical evaluation performed by the physical therapist to check the integrity of muscles and of the ligaments.
- Repeat steps d–h for every rehabilitation session (4–5 patients × 2 sessions/day × 7 days).
- At the end of the entire program, the patient is re-evaluated to determine the achieved progress.

The rehabilitation motions are performed entirely by the robotic system after the patient’s impaired limb is strapped into the attaching devices. The motion amplitudes are introduced into the user interface by an operator. The amplitudes were previously defined by a kinetotherapist based on the patient spasticity and overall health. Additionally, the interface allows speed and repetition control, parameters also given by the kinetotherapist.

#### 2.2.2. Patient Characteristics and Clinical Trials

After the development of the experimental model, the functional validation test, and the definition of the robotic rehabilitation protocol, the ethical approval for performing clinical trials was obtained in August 2019, in accordance with the Helsinki principles regarding biomedical research, and the robotic system was installed in a room at the Municipal County Hospital Cluj-Napoca within the Neurology Department.

A number of 24 patients were admitted to the clinical study, half of them with bilateral upper limb impairment (12) and the other half with right- (6) or left (6)-side hemiparesis. The physical state of the robotic system at the time of clinical study allowed only right limb rehabilitation, and the group of 24 was divided in two groups: 18 patients (12 suffering bilateral disorder and 6 suffering right hemiparesis) that performed the rehabilitation with the robotic system and 18 patients (same 12 with bilateral disorder and 6 with left arm hemiparesis) that performed classical rehabilitation performed by a kinetotherapist. Every patient from the clinical study was admitted into the hospital, evaluated before the therapy, and programmed for two rehabilitation sessions per day for a time span of 7 consecutive days. At the end of the 7 days, the patients were reevaluated to establish the results of the rehabilitation procedure.

**Figure 9:**
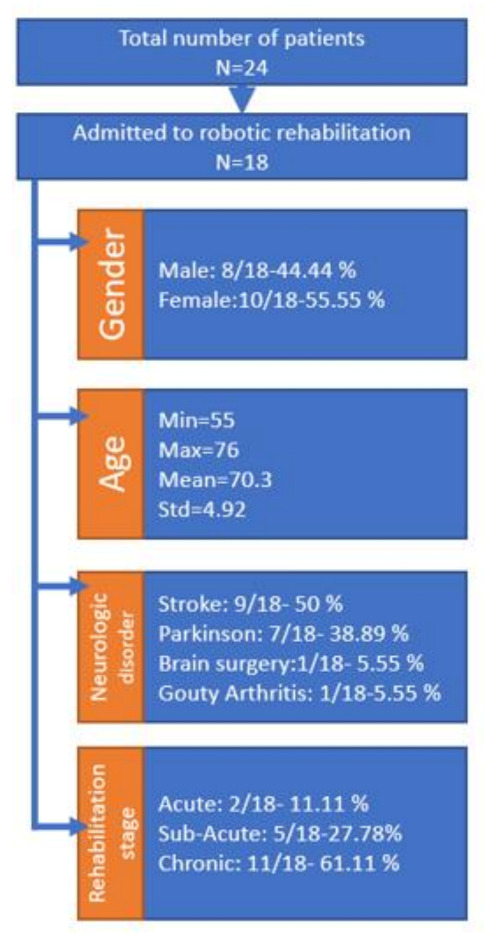
General patient data [52].

Figure 9 illustrates some of the general patient characteristics at their admittance within the clinical study. After the first evaluation of the robotic system by the clinicians, they decided that, even though the robotic system was able of performing complex rehabilitation motions, the clinical study should stick to simple motions to comply with the classical rehabilitation procedure performed by the kinetotherapist, ensuring a proper comparison of the rehabilitation results between the two approaches. During the study, each patient performed two sessions per day, each session consisting of 3 sets of 10 repetitions.

The tests were performed in hospital between October and November 2019 over a time span of 8 weeks. During the tests, each patient performed shoulder rehabilitation with ASPIRE for 35 min/day. Two therapy sessions were scheduled every day for each patient, one session in the morning and the other one in the afternoon (each session lasted about 17 min, excluding setup times).

**Figure 10:**
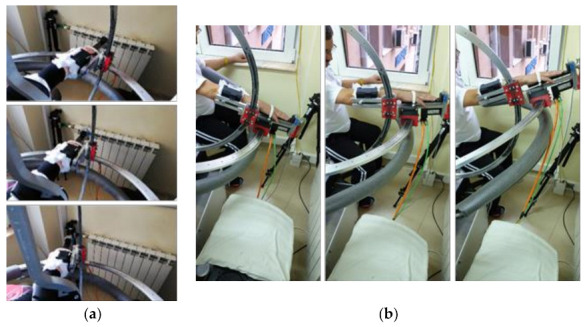
Snapshots from the experimental tests using ASPIRE: (**a**) flexion–extension motion and (**b**) adduction–abduction motion.

Snapshots from the videos recorded during the rehabilitation can be seen in Figure 10a,b.

Starting from the initial evaluation by the clinician of each admitted patient, the initial motion amplitudes were imposed with respect to the neurological disorder and specific comorbidities of each patient. The amplitudes were increased 5–10% per day, reaching the maximum amplitude on day 5 [53].

### 2.3. Questionnaire-Based System Optimization

**Figure 11:**
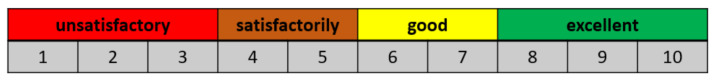
Satisfaction scale.

At the end of the rehabilitation program, each of the patients that performed rehabilitation with the robotic system were asked to complete a questionnaire regarding their experiences with the robotic system. The questions were divided based on the rehabilitation motions performed. The analyzed characteristics and their recorded values for the height adjustment module are given in Table 2, for the adduction/abduction module, are given in Table 3, and for the flexion/extension module, are given in Table 4. There are no recorded data for the pronation/supination module, since this module was not used during the rehabilitation procedure, because the classical rehabilitation protocol did not include this motion during the therapy. Each patient was asked to mark each of the characteristics with a number between 1 and 10 using the satisfaction scale given in Figure 11.

**Figure 12:**
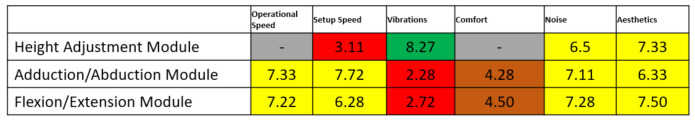
Scores obtained by each characteristic.

The score obtained by each characteristic is highlighted in Figure 12. The score for each characteristic was obtained by averaging the values given by each patient. The color code used in Figure 12 is the same from Figure 11. The characteristics highlighted in red definitely need to be improved, and the solutions for their improvements are shown in the Results section.

Using the same scale, a questionnaire was provided for the clinicians in order to evaluate the robotic system. The clinicians had different aspects to consider regarding the User Interface, the Operating Speed, the Safety, and the Amplitudes. The questionnaire was sent to 10 clinicians that also saw the robotic system while operating. The marks given by each clinician are shown in Table 5. There are some aspects that also need to be improved according to the feedback provided by the clinicians. The lowest-scoring characteristic was the user interface. This aspect is in the development stage, along with the reconfiguration of the control system. The aspects regarding the mechanical structure are discussed below; the aspects regarding the user interface and the control system are yet to be addressed before the next set of clinical trials.

## 3. Results

During the experimental tests, a series of characteristics was observed by the clinicians and by the operators and recorded to be improved. Some of the characteristics recorded were similar with the ones identified by the questionnaires. The main issue and the solution to solve every one of them is given below.

Observation 1: Times in adjusting the height mechanism were taking too long, extending the total time of the rehabilitation process.

**Figure 13:**
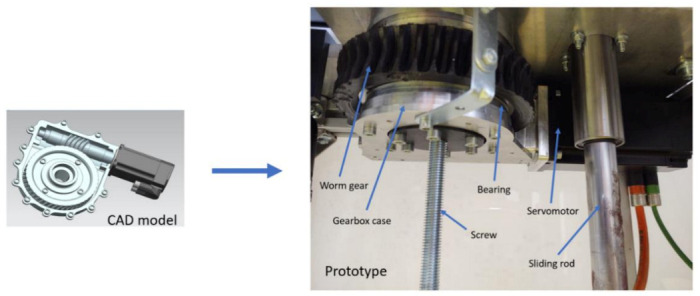
Worm gear (1:45 ratio CAD (Computer Aided Design) model and prototype).

Solution: To reduce the setup time, the height adjustment mechanism was not used during the rehabilitation; instead, a liftable chair was used. After the experimental trials, the robotic system underwent the second-stage optimization process, and the screw–nut mechanism was replaced with a ball screw mechanism, and in order to ensure the locking of the mechanism, a worm gear box was designed (Figure 13).

Observation 2: Due to the circular guides displacement, sometimes, the flexion–extension mechanism was jammed or slowed down by the patient.

**Figure 14:**
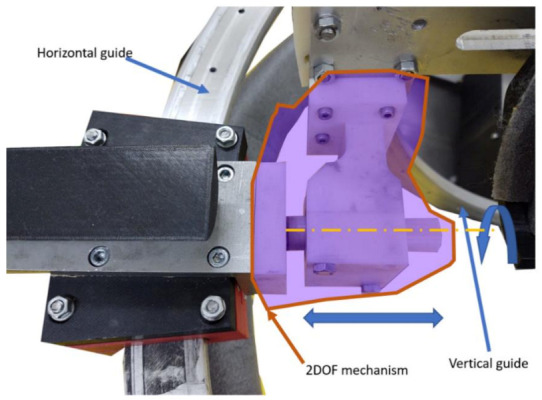
Two-Degrees of Freedom (DOF) mechanism between the circular guides.

Solution: The slides were properly greased at the beginning of the rehabilitation procedure, and a two-DOFs mechanism was used to overcome the angular displacement between the circular guides (Figure 14).

Observation 3: The shoulder anchor was problematic during the rehabilitation process.

Solution: The shoulder anchor was redesigned to eliminate any edges of the component and allow easy attachment of the shoulder. Additionally, a synthetic foam was used to cover the part where the patient was in direct contact.

Observation 4: The forearm anchor mechanism was uncomfortable for the patient.

Solution: The entire part was covered with synthetic foam, and to avoid the times needed to sterilize the component, the entire part was covered with single-use medical cotton for every patient.

Observation 5: During the use of the system, the motor of the adduction–abduction mechanism heated up.

Solution: The heating of the motor was determined to be caused by the momentum created by the weight of the mechanism transmitted through the reduction box; to overcome this situation, the same worm gear box (Figure 13) was adapted to fit this mechanism.

Observation 6: The edges of the circular guides were exposed and could hurt the patient or the operator.

Solution: The slides were covered with a soft material (synthetic foam).

**Figure 15:**
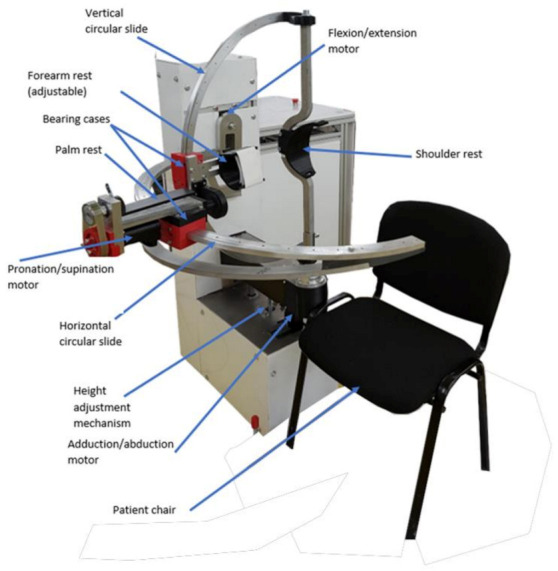
Optimized structure after the clinicians’ feedback and clinical trials.

The robotic system optimized after the clinicians’ feedback and clinical trials can be viewed in Figure 15.

## 4. Discussion

The clinical study performed on 24 patients with different neurological disorders with upper limb impairment aimed to evaluate the performance of the robotic system compared to the human therapists. The patients were split into three groups:

1. The fully robotic-assisted rehabilitation group, consisting of six patients with right limb impairment.
2. The mixed rehabilitation group, consisting of 12 patients with bilateral impairment, having their right upper limb treated with the robot and the left side with the help of a kinetotherapist.
3. The classical rehabilitation group, consisting of six patients with left limb impairment, treated by a kinetotherapist.

The patient evaluations were performed at admittance to the hospital and after the rehabilitation therapy. Besides the determination of the therapeutic efficiency of the exercises, the initial data was used as the input motion amplitudes parameters for the robotic system as individual values for each patient.

The patient evaluation consisted of two types of measurements:

(1) Ranges of motion and muscle strength—goniometry and dynamometry.
(2) Multimodal neurophysiological motor system assessment—quantitative electroencephalogram, motor conduction times, and turn/amplitude analysis [54].

Based on the size of the group, for the statistical analysis of the data, the chosen method was nonparametric testing (the Wilcoxon Matched Pairs Signed Ranks test and Mann–Whitney *U* test), with a significance threshold of *p* < 0.05.

All 24 patients performed the same therapy for seven consecutive days with two daily sessions. To perform identical exercises, which would enable a proper comparison of the therapeutic results, in this initial set of tests, only simple motions were performed: flexion/extension and adduction/abduction. The starting values for the motion amplitudes were established by the neurologists at the individual level and increased daily 5–10%, aiming to reach a full range of motion on the fifth day.

Based on their neurologic pathology, the patients were grouped into three groups: vascular, extrapyramidal, and neuromuscular.

The clinical study, which is described in detail in [53], pointed out several important conclusions on the medical side:

1. There were no statistically significant differences between the robot-assisted or physical therapist rehabilitation therapy.
2. The vascular group showed the most significant results, confirming the positive in-fluence of physical rehabilitation for stroke patients.
3. Confirming the data from other studies, some positive effects were seen for the extrapyramidal group, validating the results from other clinical studies that stated that physical exercises show benefits in Parkinson’s disease [54] and should be used in the long-term management of this pathology.

**Figure 16:**
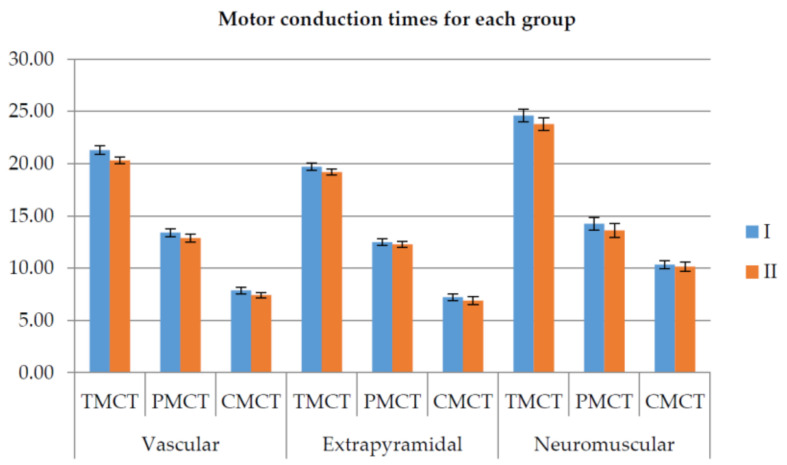
Total (TCMT), peripheral (PMCT), and central (CMCT) motor conduction time before (I—blue) and after the 7-day rehabilitation therapy (II—orange) [53].

Figure 16 illustrates the motor conduction times for the three groups measured before and after rehabilitation therapy (lower is better).

Next, the discussion will focus on the technical aspects of the clinical trials directly related to the robotic system.

No incidents were reported during the clinical trials, and the robotic system functioned within the normal functioning parameters for an average of four h/day.

One of the patients (no. 1) suffered a shoulder fracture 40 years ago, and he was unable to perform the physical rehabilitation with the robot. Another patient (the one suffering from gouty arthritis) manifested completely different from other patients, succeeding in performing the rehabilitation motion at the maximum amplitude since day 1.

Some of the patients (44.44%) used a wheelchair instead of the chair provided by the robotic system due to their general condition. Using a wheelchair during the clinical trials proved to be a challenge due to restrained space provided by the room where the robot was placed. The time spent by each patient performing the rehabilitation procedure was longer than the one spent performing manual rehabilitation, but the longer time spent performing the rehabilitation turned to be a positive aspect of the rehabilitation. Contrary to general opinion that a patient will manifest reluctance when robotic devices substitute medical personnel, all the patients were rather excited to work with the robotic system and eager to test the new technology. During the clinical trials, to create a comfortable environment for the patient, music was played in the background, this aspect proved to be a motor positive factor for the patients and a reinforcement for performing the rehabilitation exercise.

Based on the results and the feedback from the patients, the clinical trials were a real success, motivating the research team to continue in improving the rehabilitation system. At the time of the clinical trials, only simple motions were performed, but the obtained results created the premise for improving the robotic system in terms of a control system to embed more safety characteristics for the patients and for the system and developing an improved and reliable user interface.

Future works will target the extension of the robotic system functionalities via the control system to enable human–robot interaction modalities and develop a multilevel user interface that can be easily and safely used by every person, with the next set of clinical trials being scheduled for the second half of 2021.

## 5. Conclusions

The robotic system for shoulder rehabilitation ASPIRE was successfully tested in a hospital environment using patients with real neurologic disorders. The work performed with real patients revealed a significant difference from the initial tests performed in the laboratory with healthy subjects and provided a series of critical characteristics required to be improved in the development of the robotic system. ASPIRE was initially designed for patients with upper limb impairment post-stroke, but the clinical trials proved its usefulness for multiple neurologic disorders (stroke, Parkinson’s, and gouty arthritis). After the successful completion of the clinical trials, the entire robotic structure underwent a detailed analysis, and a series of improvements were made to improve the functionality of the robotic system and the quality of the patients spent during the rehabilitation task. After the design improvements were made, a new series of clinical trials are planned as soon as the COVID-19 pandemic will allow them.

## Figures and Tables

**Table 1:** Robotic devices for upper limb rehabilitation. DOF: Degree of Freedom.

Name	Targeted Area	DOF	Therapy Type	Characteristics	Stage of Development
MIT-MANUS [30]	Elbow, Wrist	5	Physical therapy	End-effector-based	Commercial
ARMin [31]	Shoulder, Elbow, Forearm, Wrist	7	Physical therapy	Exoskeleton-based	Commercial
REHAROB [32]	Shoulder & Elbow	12	Physical therapy	2 modified industrial robots	Clinical trials with low number of participants
SUEFUL-7 [33]	Shoulder & Elbow	3	Power assistance	Wheelchair mounted system	Healthy subjects test
ARMOR [34]	Shoulder, Elbow, Forearm, Wrist, Fingers	8	Physical therapy	Exoskeleton-based	Clinical trials with low number of participants
L-EXOS [35]	Shoulder, elbow	4	Physical therapy	Exoskeleton-based	Feasibility clinical study
ArmeoSpring [36]	Shoulder, Elbow, Forearm, Wrist, Fingers	7	Physical therapy	Exoskeleton-based	Commercial
ReoGo [37]	Shoulder, Elbow, Wrist	3	Physical therapy	End-Effector based	Commercial
Sophia-4 [38]	Shoulder, Elbow, Wrist	2	Physical therapy	End-effector based, cable-driven	Prototype
Pneu-WREX [39]	Shoulder, Elbow, Fingers	4 + 1	Physical therapy	Wheelchair mounted, gravity balancing orthosis	Feasibility clinical study

**Table 2:** Height adjustment module.

Characteristic /Patient	Operational Speed	Setup Speed	Vibrations	Comfort	Noise	Aesthetics
1	NA	2	8	NA	7	8
2	NA	3	9	NA	6	7
3	NA	2	8	NA	5	8
4	NA	4	9	NA	9	8
5	NA	1	8	NA	8	8
6	NA	5	9	NA	5	7
7	NA	4	8	NA	5	6
8	NA	3	8	NA	6	8
9	NA	6	8	NA	6	8
10	NA	7	8	NA	6	8
11	NA	1	8	NA	7	8
12	NA	4	9	NA	8	7
13	NA	2	9	NA	6	7
14	NA	3	8	NA	8	7
15	NA	3	7	NA	5	6
16	NA	3	8	NA	5	5
17	NA	2	9	NA	8	9
18	NA	1	8	NA	7	7

**Table 3:** Adduction/abduction module.

Characteristic /Patient	Operational Speed	Setup Speed	Vibrations	Comfort	Noise	Aesthetics
1	6	8	2	4	7	5
2	7	8	1	4	7	6
3	7	8	1	4	8	7
4	7	8	2	3	7	7
5	7	7	2	6	9	6
6	8	5	2	6	6	6
7	8	7	3	5	6	6
8	8	9	4	4	6	5
9	8	8	2	4	8	6
10	8	8	2	5	6	6
11	6	8	2	3	7	7
12	9	8	3	6	8	6
13	8	8	2	5	6	8
14	8	9	2	5	8	8
15	8	9	2	4	6	6
16	6	7	3	3	8	6
17	6	7	3	3	8	6
18	7	7	3	3	7	7

**Table 4:** Flexion/extension module.

Characteristic /Patient	Operational Speed	Setup Speed	Vibrations	Comfort	Noise	Aesthetics
1	6	8	2	4	8	8
2	6	3	3	5	8	9
3	8	6	3	5	8	9
4	8	9	4	6	9	9
5	8	7	4	2	9	8
6	7	7	3	4	8	6
7	5	6	2	4	8	6
8	6	6	2	3	7	8
9	6	5	1	4	6	8
10	6	6	2	3	8	8
11	8	8	2	4	4	7
12	8	4	3	5	5	7
13	9	7	2	5	8	6
14	9	7	3	6	8	6
15	8	6	3	4	6	6
16	8	6	3	6	5	8
17	8	6	4	5	8	8
18	6	6	3	6	8	8

**Table 5:** Marks given by the clinicians.

Characteristic /Clinician	User Interface	Operating Speed	Safety	Amplitudes
1	9	7	8	8
2	8	7	9	6
3	5	8	9	8
4	6	8	7	9
5	5	7	8	7
6	3	6	5	5
7	4	5	7	6
8	8	8	7	6
9	5	9	6	8
10	5	8	5	6
SCORE	5.8	7.3	7.1	6.9

## Data Availability

The data presented in this study are openly available in reference number [52,53].
